# Supplementary material for: Microscopic and Biochemical Hallmarks of BICD2-Associated Muscle Pathology toward the Evaluation of Novel Variants
Source: Int J Mol Sci. 2023 Apr 6;24(7):6808. doi: 10.3390/ijms24076808 (PMC10095373; doi:10.3390/ijms24076808)
Supplement: Supplementary file 1 [file ijms-24-06808-s001.zip › Supplementary Table S1.pdf]

Supplementary Table S1

BICD2 variant	Classification (ACMG criteria)	Prediction programs	conservation	Frequency in GnomAD	Reported in ClinVar database (number of entries)	Reported in the literature
p1<br><br>c.320C>T, p.(Ser107Leu) rs398123028	Pathogenic (PM1, PM2_sup, PP1, PS3, PS4_mod)	Mostly predicted as pathogenic:<br>MutationTaster: disease causing<br>PROVEAN: damaging<br>SIFT: damaging<br>Revel: 0.36 (uncertain)	Strong	-	ClinVar: pathogenic (8)	Bansagi et al., 2017<br>Unger et al., 2016<br>Rossor et al., 2015<br>Neveling et al., 2013<br>Peeters et al., 2013<br>Oates et al., 2013
p2<br><br>c.1195C>T, p.(Arg399Cys) rs200542458	Uncertain significance (BP4_sup, PM2_sup)	Conflicting predictions:<br>MutationTaster: disease causing<br>PROVEAN: neutral<br>SIFT: tolerated<br>Revel: 0.21	Moderate	0.0000159	ClinVar: uncertain significance (1), likely benign (1)	Not reported in literature
p3 and p4<br><br>c.2189G>A, p.(Arg730His) rs943974428	Uncertain significance (PM2_sup, PM1)	Mostly predicted as pathogenic:<br>MutationTaster: disease causing<br>PROVEAN: damaging<br>SIFT: damaging<br>Revel: 0.631	Strong	-	Not reported in ClinVar	Not reported in literature
p5 and p6<br><br>c.1904G>T, p.(Arg635Leu)	Uncertain significance (PM2_sup)	Mostly predicted as pathogenic:<br>MutationTaster: disease causing<br>PROVEAN: damaging<br>SIFT: damaging<br>Revel: 0.525	Strong	-	Not reported in ClinVar	Not reported in literature
p7<br><br>c.2452A>G, p.(Lys818Glu) rs200341779	Uncertain significance (BP4_mod)	Conflicting predictions:<br>MutationTaster: disease causing<br>PROVEAN: neutral<br>SIFT: damaging<br>Revel: 0.112	Moderate	0.0000322	ClinVar: likely benign (2)	Not reported in literature

NM_001003800.1

**Variants in other genes**	**Classification**	**Prediction programs**	**conservation**	**Frequency in GnomAD**	**Reported in ClinVar database**	**Reported in the literature**
p5 and p6<br><br>*FLNC*<br>c.2272G>A,<br>p.(Val758Met)<br>rs371418145	Uncertain significance	Mostly predicted as pathogenic:<br>Mutationtaster: disease causing<br>PROVEAN: damaging<br>SIFT: damaging<br>Revel: 0.506	moderate (conservation score low)	0.0000725	ClinVar: uncertain significance (5)	Janin et al., 2017 (supplement)
p7<br><br>*COL6A1*<br>c.1694G>A,<br>p.(Arg565Gln)<br>rs886057155	Uncertain significance (BP4_mod)	Mostly predicted as benign:<br>Mutationtaster: polymorphism<br>PROVEAN: neutral<br>SIFT: tolerated<br>Revel: 0.175	weak	0.000026	ClinVar: uncertain significance (4), benign (1)	Not reported in literature
