# Supplementary figures and images for: Microscopic and Biochemical Hallmarks of BICD2-Associated Muscle Pathology toward the Evaluation of Novel Variants
Source: Int J Mol Sci. 2023 Apr 6;24(7):6808. doi: 10.3390/ijms24076808 (PMC10095373; doi:10.3390/ijms24076808)

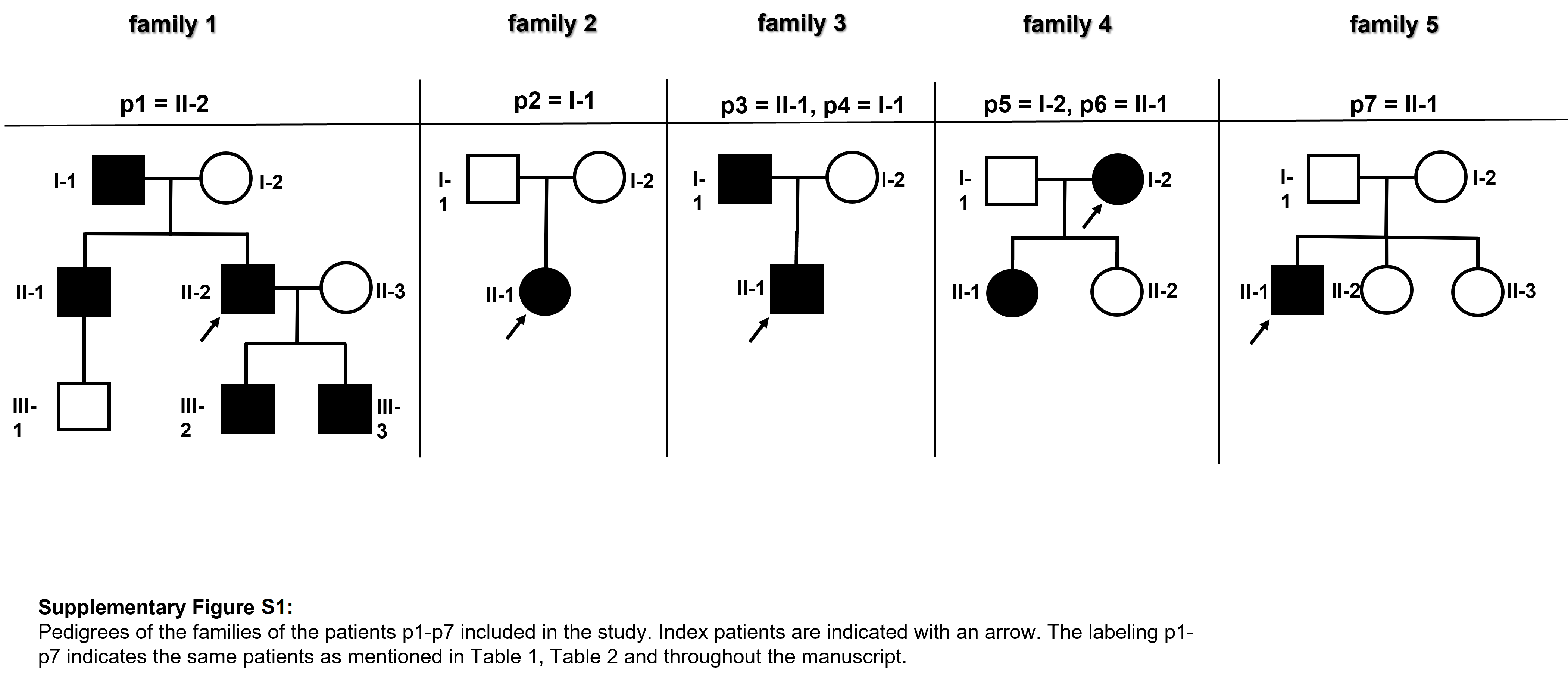

Supplement: Supplementary file 1 [file ijms-24-06808-s001.zip › ijms-2268654-Supplementary Figure S1.png]
